# The Study on the Whole Stress–Strain Curves of Coral Fly Ash-Slag Alkali-Activated Concrete under Uniaxial Compression

**DOI:** 10.3390/ma13194291

**Published:** 2020-09-25

**Authors:** Huailiang Wang, Lang Wang, Lei Li, Baoquan Cheng, Yonggang Zhang, Yuhu Wei

**Affiliations:** 1 College of Civil Engineering and Architecture, Guangxi University, Nanning 530004, China; Whuailiang@gxu.edu.cn (H.W.); wanglang@st.gxu.edu.cn (L.W.); 2 Key Laboratory of Disaster Prevention and Structural Safety of Ministry of Education, Nanning 530004, China; 3 School of civil engineering, Xi’an University of Architecture and Technology, Xi’an 710055, China; lilei@xauat.edu.cn; 4 School of Civil Engineering, Central South University, Changsha 410083, China; 5 Key Laboratory of Geotechnical and Underground Engineering of Ministry of Education, Department of Geotechnical Engineering, Tongji University, Shanghai 200092, China; demonzhangyg@tongji.edu.cn; 6 College of Civil Engineering, Anhui Jianzhu University, Hefei 230000, China; aarondamen@163.com

**Keywords:** coral aggregates, fly ash-slag alkali-activated concrete, monotonic loading method, whole stress–strain curve, elastic modulus, Poisson’s ratio

## Abstract

This study aimed to research the whole stress–strain curves of coral Fly Ash-Slag Alkali-Activated Concrete (CAAC) in different strength grades. Fly ash-slag alkali-activated concrete was used as cementing materials to produce coral aggregate concrete. A monotonic loading test was conducted on the prismatic specimens to obtain elastic modulus (*E*_c_), Poisson’s ratio (*μ*) and the constitutive relationship of CAAC under uniaxial compression. When the strain of CAAC reached the maximum value, the specimen was split and damaged rapidly. As the strength grade increased, the ratio of residual stress (*σ*_ri_) to peak stress (*σ*_0i_) decreased in the range of 0.17–0.28. The *E*_c_ of CAAC increased gradually, and *μ* increased to the peak value and then decreased. According to the test results, the constitutive equation of CAAC can be expressed by piecewise expression, which can better reflect all the experimental characteristics. It was also found that CAAC has many similar characteristics with coral concrete and lightweight aggregate concrete. To improve the strength and toughness of CAAC, some fibers, such as organic fiber, can be added to expand the application of CAAC in engineering projects.

## 1. Introduction

In recent years, with the rapid development of urban construction, the amount of cement used worldwide has surged. At the end of the last century, the global cement consumption exceeded 2.2 billion tons per year [1]. According to Elchalakani [2], the production of ordinary Portland cement reached 3.7 billion tons in 2017, which was accompanied by 3 billion tons of CO_2_ emissions, accounting for about 7% of the total global emissions. Statistics released by the European Cement Association [3] showed that the cement output of the European Union was about 170 million tons in 2016, making it one of the major cement producers in the world. In 2016, China produced more than half of the world’s cement, exceeding 2.4 billion tons. From 2015 to 2016, Australia’s cement output was nearly 10 million tons, up 5% year on year [4]. Hao et al. [5] and Cheng et al. [6] also reported that concrete is one of the most important sources of environmental impacts caused by construction activities.

The environmental problems caused by cement production in the world cannot be ignored [7].

According to the report, during cement production [8], every ton of cement can generate about 1 ton of carbon dioxide emissions, and more than 1/20 of the global carbon dioxide emissions are related to cement production [9]. Therefore, many researchers have been working to develop an alternative to ordinary Portland cement construction material in recent years.

At the same time, with the rapid development of urbanization, many high-rise buildings, highways, factories, etc. have been built, which requires a large amount of concrete [10]. Because of its low cost and long durability, concrete has become the most commonly used building material, with an annual consumption of about 30 billion tons [11]. However, the raw materials used to make concrete will lead to large amount of carbon emissions during the manufacturing, which will cause pollution to the environment [12,13]. In addition, the production of a large amount of concrete requires a lot of sand, stone, water and other raw materials; the large consumption of these raw materials will cause severe damage to the land, water resources and air quality. In other words, the environmental quality will be greatly affected. Therefore, a new type of concrete, with both economic and ecological benefits, is badly needed [14,15,16,17,18,19,20,21,22].

The emergence of geopolymer [23] is expected to highly reduce the impact of construction activities on the environment. Geopolymer is made from industrial wastes and contains no cement. Compared with ordinary concrete (OPC), the same amount of geopolymer concrete (GC) can be produced with 40% of the energy required, while CO_2_ emissions are reduced by 80% [24,25,26,27]. At present, there have been many studies on GC and alkali activated slag concrete (AASC). According to the existing research results, compared with traditional OPC, alkali-activated concrete (AAC) has similar or better performance in terms of early-age strength [28], fire resistance [29], thermal stability [30], water absorption, permeability [31,32], chloride resistance [33,34] and durability [35]. Previous studies on geopolymers mainly focused on the microstructure, but there were few studies on the practical application of geopolymers in structural members or structures [36,37,38,39,40,41,42]. Recently, some scholars have done some research on the mechanical properties of geopolymer concrete [43] and the properties of alkali-activated lightweight aggregate concrete [44]. Zbigniew et al. [45] studied the effect of geopolymer cementitious material fly ash and slag on the mechanical properties of concrete. Fabio et al. [46] studied the properties of fiber-reinforced geopolymer mortar. Wang et al. [47] investigated the performance of actively confined geopolymer concrete. Some scholars [48,49] had studied the influence of the variability of calcareous fly ash properties on the rheological properties of fresh mortar and the influence of nano-silica on the chemical durability and mechanical properties of fly ash-based geopolymer concrete.

Due to the exhaustion of land resources, the development of ocean is of great strategic significance. Construction materials and freshwater resources on an island are extremely limited, and construction on an island is limited by high upfront cost. Therefore, it is logical to use local coral aggregates to take the place of traditional building materials. Coral aggregates concrete is prepared using coral aggregates instead of natural sandstone, thus posing no damage to the natural ecological environment on the island. There are many coral reefs in the sea by islands, with major components of aragonite. The high magnesium calcite is a special type of rock–soil with a CaCO_3_ content of more than 96%. These reefs can be made into coral aggregates, providing a new engineering material for the construction of islands [50,51].

At present, research on coral concrete (CPC) mainly focuses on the mix design, durability and basic mechanical properties. In 1951, Dempsey [52] pointed out that, if the CPC were of high quality and density, it would suffer little corrosion, but it might be corroded after long-time exposure in a humid environment. In 1982, Vines [53] found that the strength structure and durability of local CPC were poor in Samoa, South Pacific. In 1989, Zhaolin [54,55] systematically studied the basic mechanical properties of CPC by preparing different types of CPC specimens. In 2012, Lei [56] found that CPC and lightweight aggregate concrete (LPC) have similar characteristics in strength development, failure mechanism and failure performance. In 2013, Yingtao [57] studied CPC and OPC with the same mix ratio and the performance differences in frost resistance and corrosion resistance. They found that fly ash or slag can be added to CPC to improve its freezing and corrosion resistance. The above research provides important reference for the application of CPC.

The combination of geopolymer binders and coral aggregates reduces the consumption of sand and cement and achieves eco-friendly concrete production. The whole stress–strain curve of uniaxial compression is the basis for studying the bearing capacity and deformation of concrete structures [58]. For the whole stress–strain curves of CPC and LPC, there have been some research results [59,60,61]. However, the whole stress–strain curve of CAAC that uses coral instead of ordinary aggregate has not been studied in detail.

In this study, fly ash-slag and coral aggregates were combined to prepare alkali-activated coral aggregates concrete with three strength grades. From the perspective of structural design, the whole stress–strain curve of CAAC was systematically studied and compared with normal weight alkali-activated concrete (NAAC) with limestone aggregates, CPC and LPC of the same strength grades. It is of great significance to provide basic constitutive relation for the application of CAAC.

## 2. Experimental Program

### 2.1. Materials

**Figure 1:**
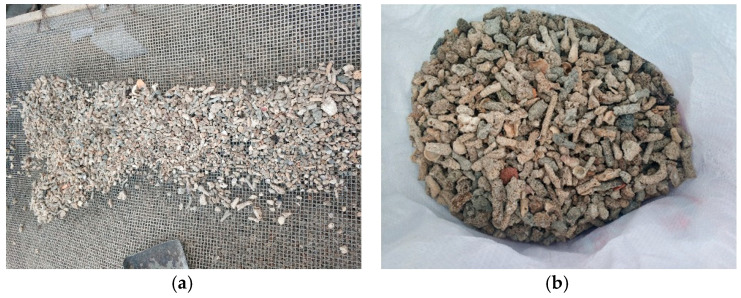
Coral aggregates for test: (**a**) broken coral; and (**b**) coral aggregates.

**Figure 2:**
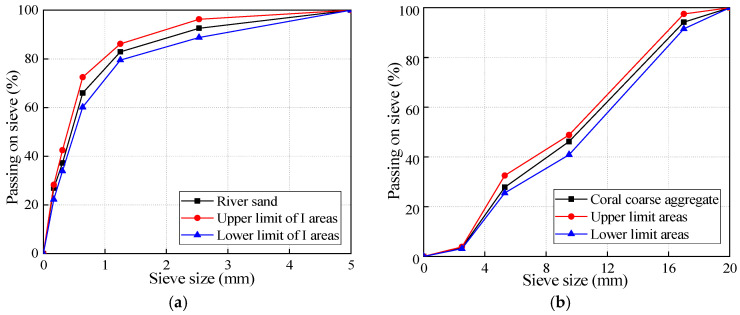
Size distribution of river sand and coral aggregates: (**a**) fine aggregates; and (**b**) coarse aggregates.

The binder materials used in this study were fly ash (FA) and ground granulated blast furnace slag (GGBFS), which obtained from China Resources mixing Station, Nanning, China. The chemical compositions and physical properties of the FA and GGBFS are listed in Table 1. Two types of coarse aggregates were used in this research project: coral aggregates (Figure 1) and limestone aggregates (Qinzhou, China). Limestone with a maximum size of 20 mm and aggregate crushing value of 23% was employed as coarse aggregates for NAAC. The coral comes from some islands and reefs in Beihai City, Guangxi Zhuang Autonomous Region, China. The coral with a maximum size of 20 mm (Figure 2b), cylinder compressive strength of 2.12 MPa and 24 h water absorption ratio of 14.2% was used as coarse aggregates for CAAC. Natural river sand (Figure 2a) with fineness modulus of 2.4 was used as fine aggregates for all concrete mixtures. The fineness modulus, specific gravity, water absorption, aggregates crushing value and cylinder compressive strength of different aggregates were determined according to Chinese National Standard GB/T 17431-1998 [62], as listed in Table 2.

### 2.2. Mixture Proportions and Specimen Preparation

The alkali activator used in this study is a mixture of NaOH solid and sodium silicate (SS) solution. For all AAC mixtures and mixtures containing additional water, the ratio of sodium silicate solution to NaOH was constant at 2.5. The chemical composition of SS solution was Na_2_O = 13.6%, SiO_2_ = 29.9% and H_2_O = 56.5% by mass. The specific gravity and the modulus of SS solution were 1.35 and 3.0, respectively. The solution of alkali activator was prepared by dissolving NaOH pellets with 99% purity in sodium silicate solution for 24 h. Potable tap water was used in all concrete mixes, and poly-carboxylic type superplasticizer (SP) with a specific gravity of 1.18 and pH 7 was used in NAAC and CAAC mixtures.

The term water/solid (W/S) ratio in AAC was defined as the ratio of the total mass of water in the mixture to the sum of the mass of blended FA-GGBFS powder material, NaOH solids and sodium silicate solids. The W/S ratio determines the strength of AAC. In this experiment, the slump value of the designed AAC was 15–22 cm. Therefore, the dosage of superplasticizer for each mixture with different W/S ratio was modified to achieve the specified slump value.

In addition, the minimum target compressive strength of CAAC should exceed 20 MPa for application to structural concrete members. In 2013, Vora et al. [63] found that the compressive strength of alkali-activated concrete decreased with the increase of water–binder ratio. Alkali activated concrete is composed of coarse aggregate, fly ash, ground granulated blast furnace slag, sodium silicate, NaOH, river sand, water and superplasticizer. According to the research of Yinfeng et al. [64] and a series of trial matches, three different strength grades of alkali-activated concrete were designed. The mix proportions are shown in Table 3.

At present, there is no standard available as a reference for the mixing procedure of AAC, especially for structural-grade lightweight AAC. In this study, FA, GGBFS and aggregates were first stirred in a drum mixer for 5 min when NAAC was made. Then, the alkali activator solution and the mixture of water and superplasticizer were gradually added to the mixer, and the stirring process lasted another 5 min. Before the preparation of CAAC, it was necessary to soak the coral aggregate in tap water to remove impurities and reduce the concentration of chloride ion and then dry them. During the preparation, the coral aggregates were firstly wetted with little sodium silicate solution and stirred for 2 min, and then FA, GGBFS and river sand were added and stirred for 2 min. Finally, the alkali activator solution, superplasticizer and water were added gradually and stirred for 5 min. All the AAC concretes were put into the steel model and vibrated on the shaking table for about 10–20 s. It is noted that sodium silicate solution treatment of coral aggregates in the casting procedure of AAC can help to improve the quality of coral aggregates particle by reducing water absorption and increasing strength.

For each strength grade mixture, three 100 mm × 100 mm × 100 mm cubes and three 100 mm × 100 mm × 300 mm prisms were cast. After casting, all specimens were covered with plastic film to prevent water loss. The specimens were taken out of the mold after 24 h and placed in a curing room at 25 ± 5 °C and relative humidity of 50–55% for 30 days.

### 2.3. Test Methods

In this study, the uniaxial compression tests were performed on RMT-201 rock and concrete mechanical test system at the Structural Laboratory of Guangxi University, Nanning, China. The maximum loading range of the testing machine was 1500 kN, with an accuracy of 0.001 kN, and the minimum loading rate was 0.001 mm/s. According to the Chinese National Standard GB/T 50081-2002 [65], the compressive stress–strain relationship test was carried out on prism specimens with size of 100 mm × 100 mm × 300 mm; at the same time, elastic modulus and Poisson’s ratio of different concrete mixtures could be estimated. The instruments used in the test were displacement transducers and recorders [66]. The load, displacement and strain were collected by corresponding data acquisition system and stored in computer during the experiment process.

**Figure 3:**
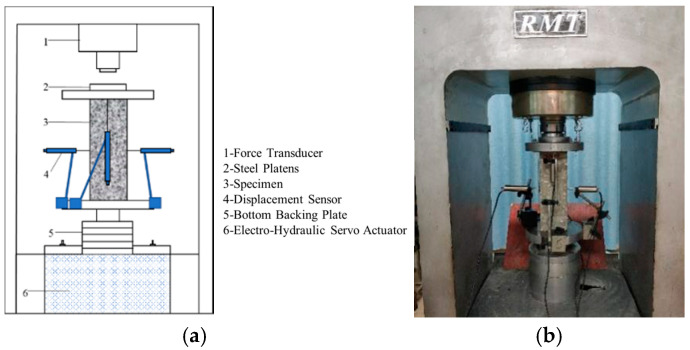
Test set-up: (**a**) schematic of setup for loading; and (**b**) test equipment.

The test equipment and the schematic diagram of the loading device are shown in Figure 3. The displacement-controlled loading mode was adopted with a loading speed of 0.002 mm/s. A linear variable differential transformer with a measurement range of ± 5 mm and precision of 400 με/mm was used to measure the axial (vertical) displacement and the lateral displacement. At the same time, to improve the accuracy of the measurement and reduce the influence of the end of the specimen and the supporting plate on the test results, the axial and lateral strains of the AACs were also measured by the strain gauges. The horizontal strain gauge was used to measure the transverse strain, while the vertical strain gauge was not only used to measure the longitudinal strain but also used to check whether the specimen was flat. Load was measured with a load sensor mounted on the top of the specimen. The load, strains, axial and lateral deformation of specimens were collected and recorded by the Donghua-DH3818Y static strain testing system (Liyang, China), and 100 data points were recorded per second. According to the cube compressive strength obtained before this study, the peak load was estimated, and the specimens were preloaded once before formal loading with 10–20% of the peak load. After formal loading, it terminated when the load dropped to 15% of the peak load [67,68].

The axial deformation in this study is the average value of the vertical LVDT and the axial strain gauges.

The complete failure process of the specimen can be seen in the loading process. During the test, it is necessary to observe and record the form and development of the crack and the shedding phenomenon on the surface of the specimen. During the loading process, the strain gauge will be damaged when the crack penetrates, so the strain gauge generally measures the data before the specimen cracks.

### 2.4. Elastic Modulus and Poisson’s Ratio

According to the American Society for Testing Materials (ASTM) [69], the elastic modulus calculation formula is as follows:

(1) $$E_{c}=\frac{\sigma_{2}-\sigma_{1}}{\varepsilon_{2}-\varepsilon_{1}}$$

where *ε*_1_ is the axial strain (0.0005); *σ*_1_ is the major principal stress; *ε*_2_ is the longitudinal strain; and *σ*_2_ is the stress corresponding to 40% of peak load.

The Poisson’s ratio, *μ*, was calculated as follows:

(2) $$\mu =\frac{\varepsilon_{t2}-\varepsilon_{t1}}{\varepsilon_{2}-\varepsilon_{1}}$$

where *ε_t_*_1_ and *ε_t_*_2_ are the transverse strains corresponding to *ε*_1_ and *ε*_2_, respectively.

## 3. Test Results and Discussion

### 3.1. Compressive Failure Mode

**Figure 4:**
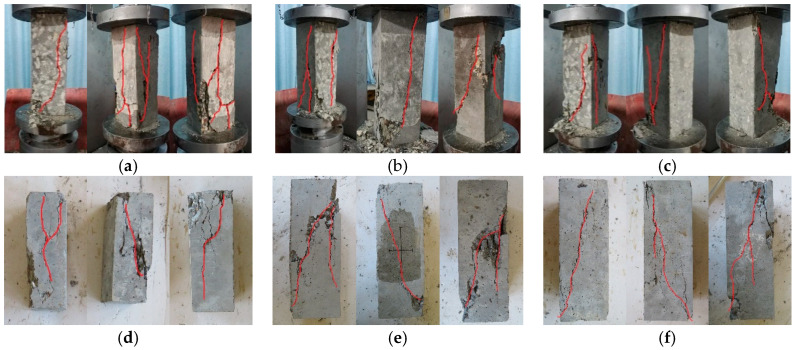
Typical failure patterns of prism specimens: (**a**) CAAC-C30; (**b**) CAAC-C40; (**c**) CAAC-C50; (**d**) NAAC-C30; (**e**) NAAC-C40; and (**f**) NAAC-C50.

The whole experimental process has three parts: (1) When the force on the specimen reached 0.10–0.20 times the peak load, the peeling phenomenon of CAAC occurred, which indicated that the specimen was in the compaction stage. (2) When the force on the specimen reached 0.6–0.85 times the peak load, small longitudinal cracks appeared on the surface of the specimen. Most cracks developed slowly and steadily. (3) When the loading continued, the crack continued to develop and extend to both ends of the specimen, and eventually led to the failure of the specimen. This indicates that the crack at this stage is not in stable development. These phenomena showed that CAAC was very brittle. With the increase of CAAC strength grade, the damage pattern was more serious and unpredictable. The failure characteristics of the NAAC and CAAC specimens were found to be similar, both with diagonal shear failure. The typical failure patterns are illustrated in Figure 4. For different strength grades of AACs, the failure modes were very similar.

### 3.2. Measured Stress–Strain Curve

**Figure 5:**
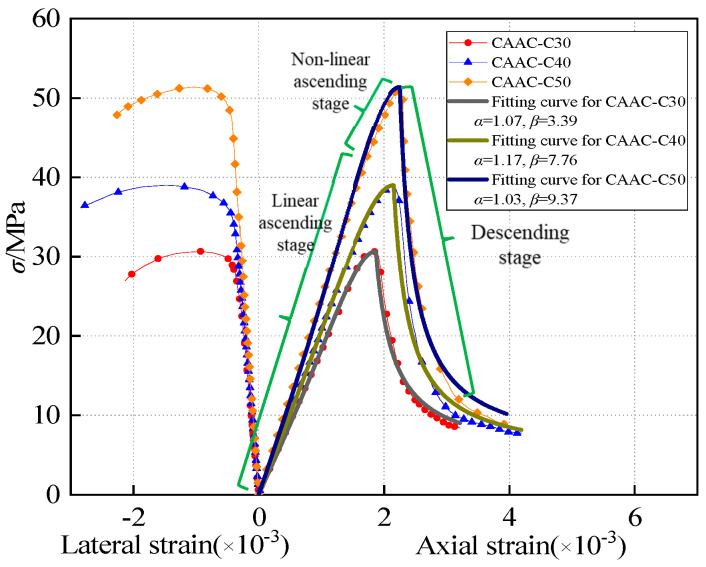
Whole stress–strain curves of CAAC in different strength grades.

**Figure 6:**
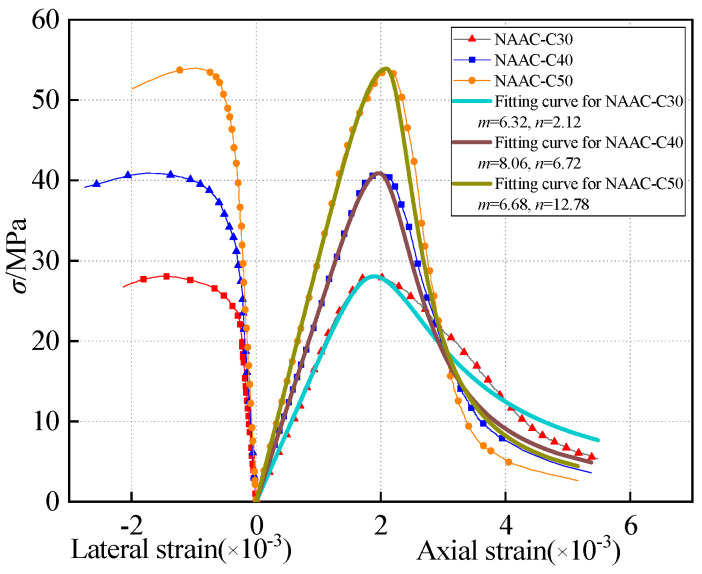
Whole stress–strain curves of NAAC in different strength grades.

Based on the load and displacement of the specimen, the stress–strain curve is plotted in Figure 5. The results show that the shape of the stress–strain curves of CAAC were very similar at different strength grades. From the beginning of loading to 0.60–0.70 times the peak load, the strain increased almost linearly and the curve began to bend. When the strain reached 0.65–0.70 times the peak strain, the turning point of the strain began to appear. The slope of the convex curve changed little, but, when the stress was close to 0.85–0.90 times the peak stress (*σ*_0_), the slope of the curve changed greatly. It can be seen that, when the stress was greater than *σ*_0_, the stress decreased rapidly, the curve dropped rapidly and the specimen was destroyed quickly and suddenly. This shows that CAAC is brittle and easily damaged when the strain is close to *σ*_0_. Meanwhile, the stress–strain curves of NAAC were similar to the curve of OPC, as shown in Figure 6.

### 3.3. Mechanical Properties

**Figure 7:**
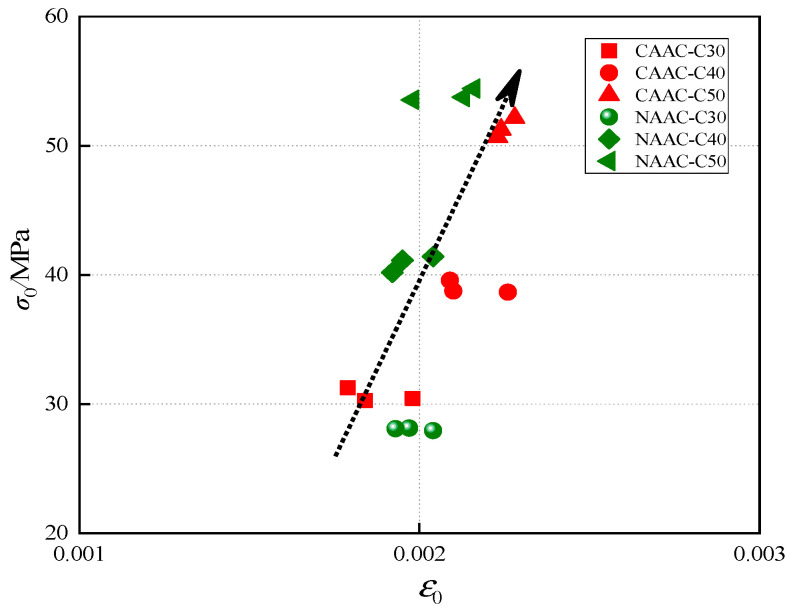
Relationship between peak stress and peak strain of CAAC and NAAC.

The peak stress (*σ*_0_), peak strain (*ε*_0_), residual stress (*σ*_r_) and ultimate strain (*ε*_u_) of CAAC and NAAC in three strength grades were measured, as listed in Table 4. The symbols *σ*_0i_, *σ*_ri_, *ε*_0i_ and *ε*_ui_ represent the mean value of *σ*_0_, *σ*_r_, *ε*_0_ and *ε*_u_ of the corresponding concrete specimens, respectively. For CAAC, the value of *σ*_ri_/*σ*_0i_ was in the range of 0.17–0.28, decreasing with the increase of strength grade, which may indicate that the damage of CAAC is more serious with the increase of strength grade. The value of *ε*_u_/*ε*_0_ was in the range of 1.72–1.94, which increased at first and then decreased as the strength grade increased. Figure 7 shows the relationship between *σ*_0_ and *ε*_0_ of CAAC and NAAC. It can be seen that *σ*_0_ and *ε*_0_ tended to increase with the increase of strength grade, which may indicate that the anti-failure ability and safety factor of CAAC and NAAC increase with the increase of strength grade [70].

### 3.4. The Whole Stress–Strain Curve of CAAC

#### 3.4.1. Establish the Whole Stress–Strain Curve of CAAC

As shown in Figure 5, the stress–strain curves of CAAC with different strength grades could be characterized by three stages: linear ascending stage, nonlinear ascending stage and descending stage. The whole stress–strain curve had its own obvious characteristics in the ascending and descending stages, respectively. Different functions were selected to fit the experimental results. By comparing with OPC and LPC, according to the research of Zhen et al. [71], the formula for the ascending stage was as follows:

(3) $$Y=ax+(5-4a)x^{4}+(3a-4)x^{5}\;(0\leq x\leq 1)$$

where $x=\varepsilon /\varepsilon_{0}$, *ε* is the strain, *ε*_0_ is the peak strain, $y=\sigma /\sigma_{0}$, *σ* is the stress, *σ*_0_ is the peak stress and *α* is the control parameter of ascending stage curve.

Figure 5 shows that, during the descending stage, the decline of CAAC was greater than that of OPC, because CAAC is more brittle than OPC. Therefore, the OPC equation found by Zhenhai et al. [72] in the descending stage is not suitable for CAAC. The descending curve needs to be consistent with the experimental results. The formula for the descending stage was as follows:

(4) $$Y=\;\frac{x}{\beta (x-1)+x}\;(x\geq 1)$$

where $x=\varepsilon /\varepsilon_{0}$, *ε* is the strain, *ε*_0_ is the peak strain, $y=\sigma /\sigma_{0}$, *σ* is the stress, *σ*_0_ is the peak stress and *β* is the control parameter of descending stage curve.

Because the stress–strain curve of NAAC is similar to that of OPC, the analytical expression of uniaxial compression of OPC in Chinese code GB/T 50010-2010 [73] was used to express the NAAC curve, as shown in Formula (5).

(5) $$\sigma =\left\{ \begin{array}{cc} (\frac{m}{\varepsilon_{0}}\varepsilon +\frac{3-2m}{\varepsilon_{0}^{2}}\varepsilon^{2}+\frac{m-2}{\varepsilon_{0}^{3}}\varepsilon^{3})f_{c} & \;0\leq x<1\\ \frac{f_{c}\varepsilon }{n\varepsilon_{0}{\left(\frac{\varepsilon }{\varepsilon_{0}}-1\right)}^{2}+\varepsilon } & \;x\geq 1 \end{array}\right.$$

where *f*_c_ is peak stress; *ε*_0_ is peak strain; *m* is a parameter of ascending stage; and *n* is a parameter of the descending stage.

**Figure 8:**
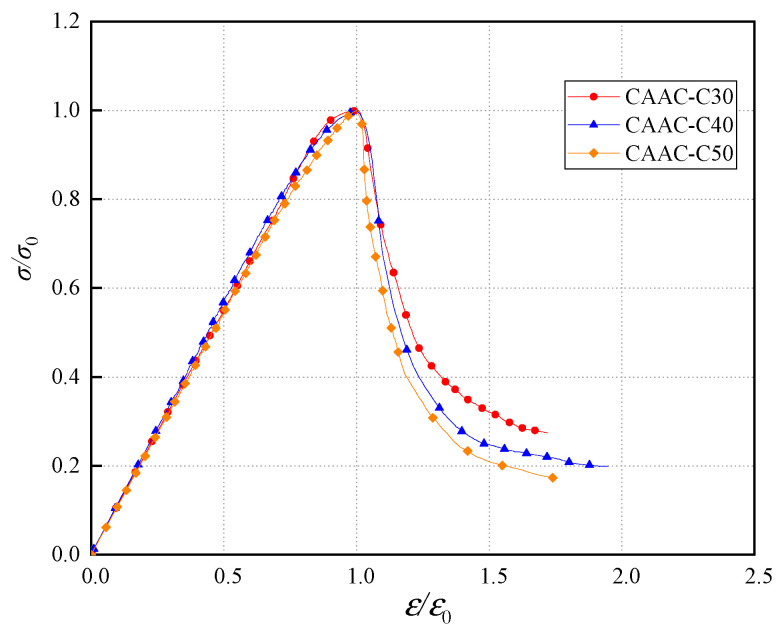
The normalized whole stress–strain curve of CAAC in different strength grades.

The normalized whole stress–strain curves of the CAAC in different strength grades is shown in Figure 8, and the curves were similar in the ascending stage. At the same time, the higher the strength grade was, the faster the decline in the descending stage was, the more serious the damage was and the smaller the relative residual stress was [50]. These trends showed that the brittleness of CAAC increased with the increase of strength grade, which was consistent with the performance of OPC. The whole stress–strain curve equation of CAAC with each strength grade has its own related parameters: *α* and *β* represent the parameters of ascending stage and descending stage, respectively, and *R*^2^ is the correlation coefficient (Table 5).

#### 3.4.2. Comparison of Whole Stress–Strain Curve for Different Types of Concrete

**Figure 9:**
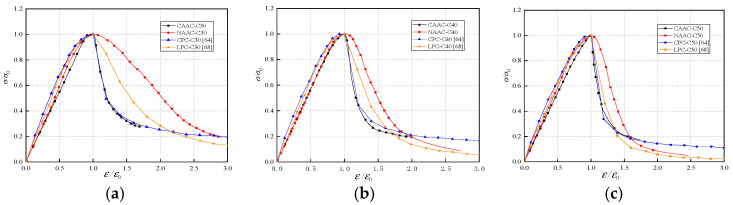
Comparison of whole stress–strain curve of CAAC, NAAC, CPC and LPC: (**a**) C30; (**b**) C40; (**c**) C50.

In Figure 9, the whole stress–strain curves of CAAC, NAAC, CPC [70] and LPC [74] are compared. It can be seen that CAAC, NAAC, CPC and LPC showed similar trends in the ascending stage, while there were significant differences in the descending stages. When the strength grade was the same, the descending stage of CAAC was the steepest, which was basically consistent with CPC, indicating that CAAC is more brittle than NAAC and LPC. This further showed that: (a) The strength of corals is lower than that of ordinary stones, volcanic rocks and shales. When the pressure reaches its maximum, the corals will quickly break. (b) The surface of coral is rough and has many pores, which increases the contact area of the interface larger. This is conducive to increasing mechanical interlock and enhancing the combination of coral aggregates and alkali-activated cementing materials [70]. The damage mainly occurs in aggregates and cementing materials, but rarely at the interface. This failure pattern indicates an increase in brittleness of CAAC.

### 3.5. Elastic Modulus and Poisson’s Ratio of CAAC

The elastic modulus (*E*_c_) and Poisson’s ratio (*μ*) of CAAC measured in the test are listed in Table 6. The results indicate that, when the strength grade increased, *E*_c_ increased gradually and *μ* increased at first and then decreased. With higher strength grade of CAAC, the relative amount of coral aggregate and the combined surface area of aggregate and cementing material were reduced, which led to the decrease of transverse restraint of concrete and the value of *μ* decreased [70].

**Figure 10:**
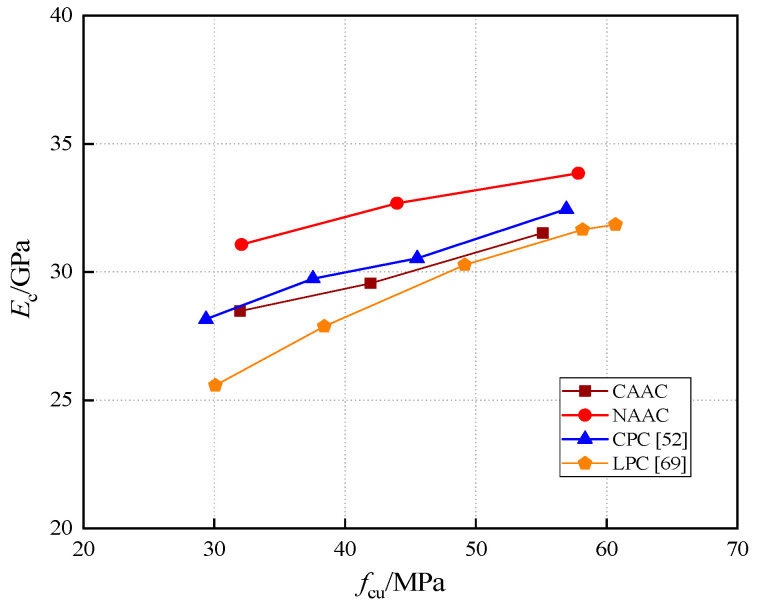
Relationship between *E*_c_ and *f*_cu_ of CAAC, NAAC, CPC and LPC.

Figure 10 shows the comparison of *E*_c_ for CAAC, NAAC, CPC and LPC. The results show that the *E*_c_ of CAAC is lower than that of NAAC and CPC, but very close to CPC, and higher than LPC. The *E*_c_ of NAAC is the largest. It can be inferred that the type of aggregates has a great influence on *E*_c_; the surface characteristics, shape, rigidity and cementing material all have great influence on *E*_c_. According to the OPC and LPC in the *E*_c_ and *f*_cu_ regression formula proposed by Faxing et al. [75], the elastic modulus of CAAC can be predicted by the following formula:

(6) $$E_{c}=3.11\times f_{\mathrm{cu}}^{0.44}$$

where *E*_c_ and *f*_cu_ represent elastic modulus (GPa) and cube compressive strength (MPa), respectively.

### 3.6. Performance Comparison

The whole stress–strain curve, *E*_c_ and *μ* of CAAC, NAAC, CPC and LPC were compared and analyzed, and the following results were obtained. When the strength grade is the same, the brittleness order for different types of concrete is as follows: CAAC > CPC > LPC > NAAC. The order of *E*_c_ is as follows: NAAC > CAAC = CPC > LPC. The brittleness and elastic modulus of CAAC limit its application in engineering construction. Therefore to expand the application scope of CAAC, it is necessary to improve its strength and toughness. Studies have shown that, to improve the strength of coral, superfine cement mortar and silicon mortar can be added on the surface of coral to form organic or inorganic covering layer to increase the density and rigidity [49]. To prevent CAAC from cracking, organic fiber can be added to CAAC [76]. These measures can produce CAAC with high strength and high toughness.

### 3.7. Potential Application of CAAC

CAAC, which uses coral as coarse aggregates, is similar to CPC in engineering properties and is expected to be applied in engineering construction. Compared with the traditional OPC, CAAC has smaller elastic modulus and larger Poisson’s ratio, thus it is suitable for foundations and infrastructures with lower rigidity requirements. Compared with LPC-based concrete, CAAC has similar mechanical properties, and coral can be used to replace some lightweight aggregates. In coastal areas, coral is often treated as waste, and it can be used as aggregates for concrete to save material. In addition, compared with OPC and LPC, CAAC has the greatest brittleness. For some important buildings and structures, this characteristic is often disadvantageous. However, CAAC is recommended for structures such as foundations and walkways, which are subjected to static loads.

## 4. Conclusions

The whole stress–strain curve of CAAC, which uses geopolymer and coral instead of cement and stone, was compared with NAAC, CPC and LPC. According to the test results, the following conclusions can be obtained:

1. Brittleness is the main cause of CAAC uniaxial compression damage. The CAAC would be divided into multiple cylinders as a splitting failure. The cracks of the specimen are mainly vertical cracks and inclined cracks. In addition, the failure of specimens usually occurs on the slope.
2. According to the whole stress–strain curve of CAAC, the shapes and characteristics vary with different strength grades. As the strength grade increases, the Poisson’s ratio (*μ*) increases at first and then decreases. The reason is that the bond surface area between coral and cementing material decreases, which leads to the decrease of lateral restraint of concrete.
3. For the whole stress–strain curves of CAAC, NAAC, CPC and LPC, there is no significant difference in the ascending stages, but there is a great difference in the descending stages. At the same strength grade, the descending stage of CAAC is the steepest compared with NAAC and CPC, which is basically consistent with CPC, indicating that CAAC is more brittle than NAAC and LPC. Superfine cement mortar and silicon mortar can be used to increase the strength of coral. It is recommended to add organic fiber to CAAC to improve the strength and toughness.

## Figures and Tables

**Table 1:** Properties and compositions of the pozzolanic materials.

Sample	Chemical Composition (%)					Blaine Fineness (cm^2^/g)	Density (g/cm^3^)	LOI (%)
	SiO_2_	Al_2_O_3_	CaO	Fe_2_O_3_	MgO			
FA	49.10	26.70	6.96	9.67	1.37	3871	2.21	2.08
GGBFS	32.28	13.80	47.85	0.74	3.73	3862	2.86	1.30

**Table 2:** Physical properties of coarse and fine aggregates.

Aggregates	Particle Size (mm)	Specific Gravity	24-h Water Absorption (%)	Fineness Modulus	Aggregates Crushing Value (%)	Cylinder Compressive Strength (MPa)
Natural river sand	≤5	2.67	1.2	2.4	-	-
Limestone aggregates	5–20	2.64	0.83	-	23	-
Coral aggregates	4–20	1.24	14.2	-	30	2.12

**Table 3:** Mixture proportions of AACs (kg/m^3^).

Mix ID	FA	GGBFS	Sodium Silicate	NaOH	River Sand	Normal Weight Aggregates	Coral Aggregates	Added Water	W/S	Superplasticizer
NAAC-C30	210	140	110	44	773	1094	-	23.1	0.29	0.9
NAAC-C40	234	156	122	49	764	1023	-	11.5	0.25	1.4
NAAC-C50	298	199	131	52	705	958	-	5.9	0.21	2.8
CAAC-C30	216	144	110	44	868	-	605	13.1	0.26	2.1
CAAC-C40	266	177	122	49	844	-	531	8.3	0.22	3.3
CAAC-C50	339	226	124	50	790	-	462	5.5	0.18	4.8

**Table 4:** Measured mechanical properties of CAAC and NAAC in three strength grades.

No.	*σ*_0_	*σ*_0i_	*S*_1_	*σ*_r_	*σ*_ri_	*S*_2_	*σ*_ri_/*σ*_0i_	*ε*_0_	*ε*_0i_	*P_1_*	*ε*_u_	*ε*_ui_	*P*_2_	ε_ui_/*ε*_0i_
**CAAC** **−30**	30.29	30.66	0.52	8.52	8.41	0.10	0.28	0.00184	0.00187	0.00010	0.00318	0.00321	0.00006	1.72
	31.25			8.38				0.00179			0.00328			
	30.44			8.33				0.00198			0.00317			
**CAAC** **−40**	38.75	39.00	0.50	7.57	7.76	0.28	0.20	0.00210	0.00215	0.00010	0.00416	0.00417	0.00005	1.94
	38.67			7.63				0.00226			0.00422			
	39.58			8.08				0.00209			0.00413			
**CAAC** **−50**	51.25	51.37	0.75	8.93	8.89	0.11	0.17	0.00224	0.00225	0.00003	0.00387	0.0039	0.00007	1.76
	52.17			8.76				0.00228			0.00398			
	50.69			8.97				0.00223			0.00400			
**NAAC** **−30**	28.14	28.06	0.10	5.35	5.41	0.07	0.19	0.00197	0.00198	0.00003	0.00542	0.00537	0.00006	2.71
	27.95			5.40				0.00204			0.00538			
	28.09			5.48				0.00193			0.00531			
**NAAC** **−40**	41.42	40.90	0.65	3.69	3.61	0.11	0.088	0.00204	0.00201	0.00004	0.00569	0.00573	0.00005	2.85
	40.18			3.49				0.00203			0.00579			
	41.13			3.65				0.00196			0.00571			
**NAAC** **−50**	54.42	53.91	0.45	2.52	2.63	0.11	0.049	0.00216	0.00209	0.00010	0.00509	0.00516	0.00006	2.46
	53.76			2.64				0.00213			0.00518			
	53.55			2.73				0.00198			0.00521			

Note: *σ*_0_ is peak stress, *σ*_r_ is residual stress, *ε*_0_ is peak strain and *ε*_u_ is ultimate strain; *σ*_0i_, *σ*_ri_, *ε*_0i_ and *ε*_ui_ are mean values of *σ*_0_, *σ*_r_, *ε*_0_ and *ε*_u_ of the corresponding concrete specimens, respectively; *S*_1_ and *S*_2_ are the standard deviations of peak stress and residual stress, respectively; and *P*_1_ and *P*_2_ are the standard deviations of peak strain and ultimate strain, respectively.

**Table 5:** Relevant parameters of whole stress–strain curve of CAAC at each strength grades.

No.	*α*	$R_{1}^{2}$	*β*	$R_{2}^{2}$
CAAC-C30	1.07	0.998	3.39	0.958
CAAC-C40	1.17	0.999	7.76	0.936
CAAC-C50	1.03	0.997	9.37	0.969

Note: $R_{1}^{2}$ is the correlation of parameter *α* in the ascending stage; $R_{2}^{2}$ is the correlation of parameter *β* in the descending stage; and the closer the value of *R*^2^ is to 1, the better are the fitting results.

**Table 6:** Elastic modulus and Poisson’s ratio of CAAC in different strength grades.

No.	*f*_cu_ (MPa)	*f*_c_ (MPa)	*E*_C_ (GPa)	*μ*
CAAC-C30	31.97	30.66	28.48	0.211
CAAC-C40	41.96	39.00	29.56	0.232
CAAC-C50	55.13	51.37	31.82	0.224

*f*_cu_, cube compressive strength; *f*_c_, prism axial compressive strength.

## Abbreviations

CAAC	coral fly ash-slag alkali-activated concrete
NAAC	normal weight alkali-activated concrete
CPC	coral concrete
OPC	ordinary concrete
LPC	lightweight aggregate concrete
AAC	alkali-activated concrete
FA	fly ash
GGBFS	ground granulated blast furnace slag
